# Inequalities in reported use of cervical screening in Estonia: results from cross-sectional studies in 2004–2020

**DOI:** 10.1186/s12905-022-02123-z

**Published:** 2022-12-24

**Authors:** Maria Suurna, Madleen Orumaa, Inge Ringmets, Kersti Pärna

**Affiliations:** 1 Department of Epidemiology and Biostatistics, National Institute for Health Development, Hiiu 42, 11619 Tallinn, Estonia; 2 Department of Research, Cancer Registry of Norway, Oslo, Norway; 3 Institute of Family Medicine and Public Health, University of Tartu, Ravila 19, 50411 Tartu, Estonia

**Keywords:** Pap smear, Cervical cancer, Trends, Screening, Prevention, Inequality

## Abstract

**Background:**

Despite the national cervical cancer (CC) screening program established in 2006, the CC incidence in Estonia in 2020 was still one of the highest in Europe. To better understand the possible barriers among women, the aim of this study was to describe the inequalities in the Pap smear uptake trend in 2004–2020 and to analyse the associations between different factors in Estonia.

**Methods:**

Weighted data of 25–64-year-old women (N = 6685) from population-based cross-sectional studies of Health Behaviour among Estonian Adult Population in 2004–2020 was used. Linear trends in uptake of Pap smear over time were tested using the Cochrane-Armitage test. Binary logistic regression with interactions was performed to analyse associations between the uptake of Pap smear and sociodemographic, socioeconomic, health-related and lifestyle factors. Crude and adjusted odds ratios with 95% confidence intervals were calculated.

**Results:**

Prevalence of lifetime uptake of Pap smear increased in 2004–2020 from 50.6 to 86.7% (*P* < 0.001). From 2004 to 2020, uptake of Pap smear increased significantly among women aged 25–34, 35–44, 45–54 and 55–64, in both ethnicity groups and among women with basic, secondary and higher education (*P* < 0.001). The gap in Pap smear uptake increased between Estonians and non-Estonians but decreased between education levels over time. Lower lifetime uptake of Pap smear was associated from sociodemographic factors with younger age, being non-Estonian and single, from socioeconomic factors with lower educational level and unemployment, from health indicators with higher body mass index indicating overweight and obesity, presence of chronic disease and depressiveness, and from lifestyle factors with non-smoking.

**Conclusions:**

Although Pap smear uptake among 25–64 year old women increased significantly in Estonia in 2004–2020, inequalities were found indicating an opportunity for development of targeted CC prevention strategies.

## Introduction

A Pap smear (abbreviated from Papanicolau test) is a cervical cytology test used to identify cell abnormalities that may indicate pre-cancerous state which may precede to cervical cancer (CC). These cell abnormalities are relatively easy to treat to prevent further progression; hence, in countries with a well-organized CC screening program both CC incidence and mortality are decreasing [1–4]. However, this has not been the case in Estonia where, despite the national CC screening program established in 2006, the CC incidence in 2020 was still one of the highest in Europe (Estonia: 27.5 per 100,000 vs. European Union mean rate: 12.8 per 100,000) [5]. The stable incidence and mortality trends [6–8] indicate the low effectiveness of organized screening program [7]; however, it must be highlighted that without screening program, the incidence is projected to be much higher [6].

In Estonia until 2021, women aged 30–55 with health insurance (ca 90%) were invited every five years to participate in an organized CC screening with a possibility to give a free of charge Pap smear regardless of their screening history. Since 2021, the CC screening target group was expanded to the age of 65, women without health insurance were included, and the HPV test was introduced as the primary test. Still, several challenges on the stakeholder level, such as high prevalence of opportunistic screening, lack of screening program quality assurance, are contributing to the high CC incidence.

A recent Estonian study found, that over half of the women with confirmed CC diagnosis did not have a Pap smear at least seven years before the disease, indicating the protective effect of having at least one Pap smear over the last seven years before the disease [9]. Earlier Estonian studies have reported, consistent with the results of national and international studies, that lower education, unemployment, low physical activity, obesity, and smoking have an unfavourable effect on Pap smear uptake [9–11]. Previous Estonian studies investigating the factors related to screening attendance are, however, over 15 years old [10, 11], or using registry data only [9], excluding valuable self-reported information. To increase CC screening attendance in Estonia it is necessary to improve the knowledge base on inequalities in Pap smear uptake.

This study was based on the theoretic framework of social determinants of cancer [12] where we focused on the effect of social and behavioural factors on the use of health care services.

The aim of this study was (1) to describe the inequalities in the Pap smear uptake trend in 2004–2020, and (2) to analyse the associations between Pap smear uptake and sociodemographic, socioeconomic, health-related and lifestyle factors.

## Methods

### Data source

This study used data from the Estonian Health Behaviour among Estonian Adult Population survey, which is a population-based cross-sectional study conducted every two years since 1990. The main topics of the self-completed questionnaire (postal, since 2016 also online) cover various questions regarding the health status and health behaviour as well as the socioeconomic status. Every second survey includes questions regarding women’s health, including Pap smear uptake. Questionnaires were in Estonian and Russian, and since 2016 also in English.

Each study year consisted of an initial sample of 5000 individuals. In 2004 and 2008, a simple random sample was selected, while in 2012–2020 a nationally representative stratified random sample of individuals from the Estonian population aged 16–64 was ordered from the Estonian Population Register. The methodology and general study procedures have been similar across the years.

### Study population and included variables

In this study, women aged 25–64 years who participated in the survey in 2004–2020, and who had answered the question “Have you ever had a Pap test (smear)?” were included. The response rates of women at this age were 71.5% in 2004, 69.3% in 2008, 70.2% in 2012, 66.4% in 2016 and 56.8% in 2020, and almost all women replied to the question on Pap smear uptake (96.3%).

The sociodemographic and socioeconomic variables used in the analysis were age (age groups: 25–34, 35–44, 45–54, 55–64 years), nationality (Estonian or non-Estonian), marital status (single, married/living with a partner, and divorced/separated/widowed), highest graduated educational level (basic, secondary, or higher education) as well as employment status (employed, not working, unemployed), and having valid health insurance (yes or no). Nationality was determined by self-assessment and classified into two groups: (1) Estonian and (2) non-Estonian (mainly Russians). Highest level of education completed was categorized as (1) basic (primary up to 6 years, basic up to 9 years, basic with vocational), (2) secondary (secondary, vocational secondary), and (3) higher (vocational higher, bachelor/master/doctoral) education. The not working category included students, those unable to work, retired, and unpaid household labour.

Health-related variables were self-rated health (good, average, poor), self-reported chronic illnesses or health problems (no or yes) and suffering from depression in the past 30 days the latter categorized into two groups: (1) no (not at all), and (2) yes (more than before/no more than before). Body mass index (BMI) was calculated based on self-reported weight and height [body weight (kg)/height^2^ (m)], and three groups were formed: (1) normal weight or less (BMI < 25.0 kg/m^2^), (2) overweight (BMI 25.0–29.9 kg/m^2^) and (3) obese (BMI ≥ 30.0 kg/m^2^). Information on self-perceived health was collected by asking women “How would you assess your current state of health? “ and grouped into three categories (1) good (good/rather good), (2) average (average), and (3) poor (rather poor/poor).

Lifestyle variables were leisure time physical activity, alcohol use, and smoking status. Women exercising at least once a week were considered active, less than once a week inactive, and women not able to exercise due to an injury or illness were marked as unable to exercise. Alcohol use was assessed by asking women whether they have ever drunk more than six units of alcohol in a single session (never, less than once a month, once a month, once a week or more often). According to the smoking status, women were divided into the following categories: (1) never, (2) past, (3) occasional, and (4) daily smoker.

### Statistical analyses

To reduce bias caused by different response rates in different study years (i.e. younger women being under-represented) poststratification weights (based on 5-year age groups) were used to compensate unit non-response (non-returned or unfilled questionnaires).

The proportion of women who reported ever having gotten a Pap smear was calculated for each study year, and linear trends in uptake were tested using the Cochran-Armitage test.

To analyse associations between the Pap smear uptake (yes vs. no) and the sociodemographic, socioeconomic, health-related and lifestyle variables, logistic regression was used to calculate both crude and adjusted odds ratios (OR) with a 95% confidence intervals (CI). Interactions between study year and all explanatory variables in the model were tested one at a time. Interaction between study year and age was included in the final model as only statistically significant interaction term. The final model was adjusted for all explanatory variables under investigation. The *P*-values of Wald chi-squared tests were presented for the adjusted logistic regression model to indicate whether the characteristic was statistically significant predictor of the outcome variable (lifetime uptake of Pap smear) or not. For all analyses, the significance level was set at *P* < 0.05.

All data was analysed using the StataMP software version 17.

## Results

Table 1 summarizes the prevalence of sociodemographic, socioeconomic, health-related and lifestyle factors among 25–64-year-old women study sample (N = 6685). Aside from age group and nationality, changes in the population distribution over the waves differed significantly (Table 1).

**Table 1:** Baseline characteristics (%) of 6685 women aged 25–64 in surveys in 2004–2020

Characteristic	Study years					*P* value
	2004	2008	2012	2016	2020	
	N = 1385	N = 1333	N = 1434	N = 1422	N = 1111	
Age group						0.396
25–34	25.3	25.0	24.4	25.6	23.8	
35–44	25.9	25.5	24.6	24.4	24.9	
45–54	26.6	27.2	25.7	24,1	25.5	
55–64	22.2	22.3	25.4	25.8	25.8	
Nationality						0.055
Estonian	67.7	68.3	71.0	71.3	72.1	
Non-Estonian	32.3	31.7	29.0	28.7	27.9	
Marital status						< 0.001
Single	9.4	11.0	13.8	11.5	13.8	
Married/living with partner	68.4	69.0	67.5	73.0	69.0	
Widowed/divorced	22.1	20.0	18.6	15.6	17.2	
Education						< 0.001
Basic	11.9	8.4	7.4	9.2	8.6	
Secondary	64.4	58.5	55.9	50.1	42.7	
Higher	23.8	33.1	36.7	40.7	48.7	
Employment						< 0.001
Employed	72.9	78.5	71.8	75.6	83.9	
Not working	22.4	19.3	21.5	19.5	12.6	
Unemployed	4.7	2.2	6.7	4.9	3.5	
Health insurance						< 0.001
Yes	92.7	95.1	95.2	95.6	96.7	
No	7.3	4.9	4.8	4.5	3.3	
BMI						
Normal	53.4	49.6	52.6	52.1	53.4	0.045
Overweight	29.9	28.8	27.0	28.2	26.2	
Obese	16.7	21.6	20.5	19.7	20.4	
Self-rated health						< 0.001
Good	38.5	48.1	52.6	54.5	59.7	
Average	50.2	42.9	36.8	35.8	30.9	
Poor	11.3	9.1	10.6	9.7	9.4	
Chronic illness						0.008
No	47.1	50.5	52.3	51.4	54.2	
Yes	52.9	49.5	47.7	48.6	45.8	
Suffering from depression in the last 30 days						< 0.001
No	27.9	31.1	35.6	40.7	43.1	
Yes	72.2	68.9	64.5	59.3	56.9	
Physical activity						< 0.001
Active	39.3	49.1	50.1	57.1	59.2	
Inactive	52.3	44.2	42.3	36.0	31.9	
Not able to exercise	8.4	6.7	7.6	7.0	8.8	
Alcohol ≥ 6 units						0.007
Never	66.4	69.3	71.1	64.6	70.1	
< Once a month	21.5	19.7	18.5	21.1	20.4	
Once a month	7.9	6.5	6.3	9.3	5.4	
≥ Once per week	4.2	4.6	4.1	5.1	4.2	
Smoking						< 0.001
Never	51.0	53.3	53.0	53.4	54.5	
Past	19.4	21.4	22.0	25.2	26.9	
Occasionally	7.7	7.1	6.9	6.1	6.2	
Daily	22.0	18.2	53.0	25.2	12.5	

**Fig. 1:**
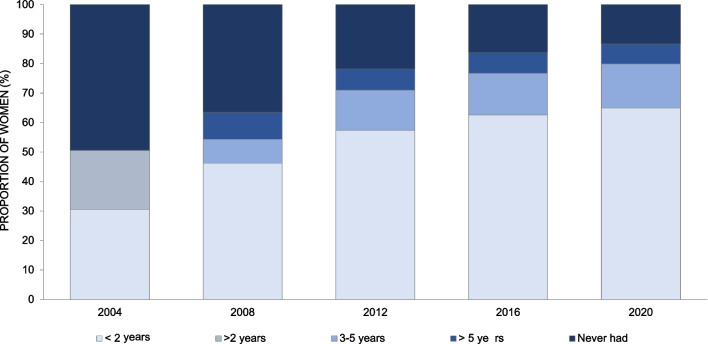
Time since most recent Pap smear. Distribution of answers to the question “When did you have your last Pap smear?” in surveys of Health Behavior among Estonian Adult Population by study year. Answer options in 2004 differed from the answer options in 2008–2020: in 2004 (1) “in the past 12 months” (2) “1–2 years ago” (3) “more than 2 years ago”. In 2008–2020 (1) “in the past 12 months”, (2) “1–2 years ago”, (3) “3–5 years ago”, (4) “more than 5 years ago”

In 2004, 49.4% of the women reported that they have never had a Pap smear, and 30.5% of women said they had it less than two years ago (Fig. 1). Since 2008, the majority of women in each survey wave reported having a Pap smear less than two years ago and this proportion has significantly increased in each survey year, reaching up to 65% in year 2020.

**Fig. 2:**
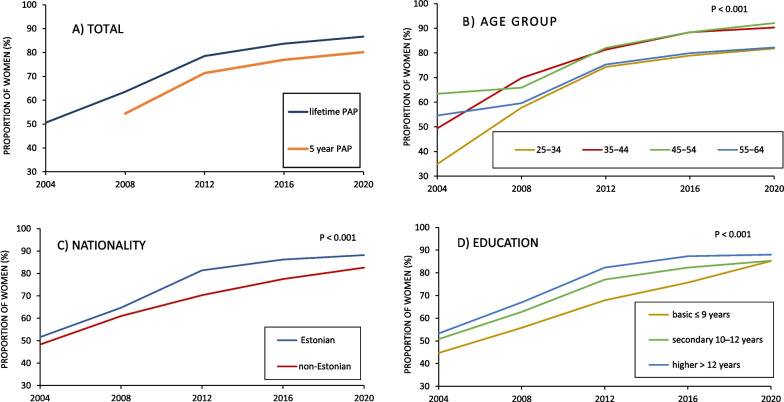
Proportion of women who had at least one Pap smear during the lifetime. **A** by total Pap smear uptake, **B** by age group, **C** by nationality, and **D** by education in Estonia from 2004 to 2020. All trend analyses were statistically significant (*P* < 0.001)

A statistically significant positive trend (*P* < 0.001) in Pap smear uptake was found from 2004 to 2020 in total, as well as across all the age groups, among Estonians and non-Estonians, and across all education levels (Fig. 2). The proportion of women who reported having had a Pap smear at some point in their life increased from 50.6 in 2004 to 86.7% in 2020 and the proportion of women who had their last Pap smear less than five years ago increased from 54.1 in 2008 to 80.1% in 2020 (Fig. 2A).

In 2004, the lifetime uptake of Pap smear was lowest among women in the youngest age group (34.7%) and highest among women in the 45–54 age group (63.4%) (Fig. 2B). Except for 2004, the lifetime uptake of Pap smear has been constantly higher among women in age groups 35–44 and 45–54, reaching over 85% in 2020, and constantly lower among women in age groups 25–34 and 55–64, but still reaching over 80% in 2020.

The gap within lifetime Pap smear uptake between Estonians and non-Estonians has increased since 2004 (Fig. 2C). While in 2004, 51.6% of Estonians and 48.3% of non-Estonians included in the study reported ever having had a Pap smear, the proportions in 2020 were 88.2% and 82.6%, respectively. The largest variation in uptake was in 2012 when 81.4% of Estonians and 70.3% of non-Estonians reported ever having had a Pap smear.

The lifetime uptake of Pap smear has always been lower among women with basic and secondary education than among women with the highest level of education, but the gap decreased over the study period (Fig. 2D). While the lifetime Pap smear uptake among women with higher education increased from 53.3 in 2004 to 88.0% in 2020, the uptake among women with the lowest education level increased from 44.7 in 2004 to 85.2% in 2020.

Adjusted logistic regression models showed that the odds of ever having a Pap smear was significantly associated with women’s age, nationality, marital status, educational level, employment status, BMI, having chronic illnesses, suffering from depression, physical activity, and smoking status (Table 2). No significant association was found between ever having had a Pap smear and having valid health insurance, self-rated health status or ever drinking six units of alcohol at once.

**Table 2:** Odds ratios of a lifetime uptake of Pap smear (yes vs. no)

Characteristic	Crude OR (95% CI)	Fully adjusted OR (95% CI)^a^	*P* value
Year & Age group^b^			0.010
2004			
25–34	1	1	
35–44	1.84 (1.34–2.53)	2.03 (1.42–2.90)	
45–54	3.26 (2.39–4.45)	4.10 (2.83–5.94)	
55–64	2.26 (1.66–3.08)	2.51 (1.72–3.68)	
2008			
25–34	1	1	
35–44	1.71 (1.24–2.37)	1.66 (1.16–2.36)	
45–54	1.41 (1.04–1.91)	1.67 (1.18–2.37)	
55–64	1.08 (0.78–1.48)	1.16 (0.79–1.69)	
2012			
25–34	1	1	
35–44	1.51 (1.04–2.20)	1.38 (0.94–2.05)	
45–54	1.59 (1.09–2.30)	1.69 (1.13–2.53)	
55–64	1.05 (0.74–1.48)	1.11 (0.75–1.64)	
2016			
25–34	1	1	
35–44	2.05 (1.34–3.14)	2.02 (1.29–3.18)	
45–54	2.04 (1.34–3.10)	2.13 (1.37–3.30)	
55–64	1.07 (0.74–1.53)	1.19 (0.79–1.78)	
2020			
25–34	1	1	
35–44	2.09 (1.24–3.52)	2.07 (1.20–3.58)	
45–54	2.69 (1.58–4.58)	2.75 (1.55–4.87)	
55–64	1.04 (0.67–1.62)	1.17 (0.73–1.89)	
Nationality			< 0.001
Estonian	1	1	
non-Estonian	0.70 (0.62–0.78)	0.66 (0.58–0.76)	
Marital status			< 0.001
Single	1	1	
Married/living with partner	1.77 (1.51–2.08)	2.11 (1.74–2.56)	
Widowed/divorced	1.62 (1.33–1.95)	2.08 (1.64–2.63)	
Education			0.003
Basic	1	1	
Secondary	1.31 (1.09–1.56)	1.28 (1.01–1.61)	
Higher	2.01 (1.66–2.44)	1.51 (1.78–1.95)	
Employment			0.004
Employed	1	1	
Not working	0.69 (0.60–0.78)	0.87 (0.73–1.03)	
Unemployed	0.58 (0.45–0.74)	0.62 (0.46–0.84)	
Health insurance			0.320
Yes	1	1	
No	60.6 (0.48–0.76)	0.86 (0.63–1.16)	
BMI			0.044
Normal	1	1	
Overweight	0.94 (0.82–1.06)	0.86 (0.73–0.99)	
Obese	0.92 (0.80–1.07)	0.82 (0.69–0.98)	
Self-rated health			0.410
Good	1	1	
Average	0.77 (0.69–0.86)	0.90 (0.77–1.06)	
Poor	0.80 (0.66–0.96)	0.97 (0.74–1.27)	
Chronic illness			0.015
No	1	1	
Yes	1.06 (0.95–1.18)	1.21 (1.04–1.40)	
Suffering from depression in the last 30 days			0.010
No	1	1	
Yes	0.95 (0.84–1.06)	1.20 (1.04–1.38)	
Physical activity			< 0.001
Active	1	1	
Inactive	0.55 (0.49–0.62)	0.69 (0.61–0.79)	
Not able to exercise	0.61 (0.50–0.74)	0.70 (0.54–0.91)	
Alcohol consumption ≥ 6 units at once			0.559
Never	1	1	
< Once per month	0.97 (0.85–1.12)	1.09 (0.92–1.29)	
Once per month	0.95 (0.77–1.18)	1.07 (0.82–1.40)	
≥ Once per week	0.88 (0.67–1.14)	0.88 (0.65–1.20)	
Smoking			0.001
Never	1	1	
Past	1.09 (0.95–1.26)	1.10 (0.94–1.30)	
Occasionally	0.83 (0.67–1.04)	0.89 (0.69–1.16)	
Daily	0.61 (0.53–0.71)	0.73 (0.61–0.88)	

^a^ Adjusted for all variables in the table (age group, nationality, marital status, education, employment, health Insurance, BMI, self-rated health, chronic illness, feeling depressed, physical activity, alcohol consumption ≥ 6 units at once, smoking) and study year

^b^ In the final model only statistically significant interaction term is presented

In adjusted logistic regression model, generally, women in the older age groups were significantly more likely to have a Pap smear than women in the youngest age group (25–34) almost in all study years. Only the difference of having a Pap smear between the youngest (25–34) and the oldest age group (55–64) was statistically significant in 2004, but not in the following study years. Compared to Estonians, non-Estonians were less likely to have had a Pap smear (OR 0.66; 95% CI 0.58–0.76). Compared to single women married/living with a partner and widowed/separated/divorced women had significantly higher odds of getting a Pap smear (OR 2.11; 95% CI 1.74–2.56 and OR 2.08; 95% CI 1.64–2.63, respectively). Compared to women with basic education, the ones with secondary education had 28% and the ones with higher education 51% higher odds for undergoing a Pap smear. Unemployed women had significantly lower odds in the lifetime uptake of a Pap smear compared to the employed ones (OR 0.62; 95% CI 0.46–0.84).

Overweight and obese women reported a slightly lower likelihood of the lifetime Pap smear uptake than women with normal weight or less (OR 0.86; 95% CI 0.73–0.99 and OR 0.82; 95% CI 0.69–0.98, respectively). Women with chronic illness were more likely to have a Pap smear than women without chronic illnesses (OR 1.21; 95% CI 1.04–1.40). Compared to women not suffering from depression, depressed women had significantly higher odds to have Pap smear (OR 1.20; 95% CI 1.04–1.38). Leisure time physical activity was a significant predictor of the Pap smear uptake: physically inactive women were 31% and women not being able to exercise were 30% less likely to undergo a Pap smear test than physically active women. The odds of giving a Pap smear during the entire lifetime were 27% lower for daily smokers (OR 0.73; 95% CI 0.61–0.88), than those women who have never smoked.

## Discussion

This study assessed the Pap smear uptake trends in Estonia from 2004 until 2020 and the factors associated with the uptake, using data from five waves of a large national cross-sectional population-based survey. The lifetime uptake of Pap smears increased significantly among all age groups, among Estonians and non-Estonians as well as across all education levels. Over the 16-year study period, the uptake gap between age groups and education levels decreased but the inequality between Estonians and non-Estonians increased. Pap smear uptake was influenced by several sociodemographic, socioeconomic, health-related and lifestyle variables, among which education and marital status had the strongest impact on Pap smear uptake.

The present study showed that lifetime Pap smear uptake increased significantly among 25–64-year-old women in Estonia during 2004–2020. Similarly, a Lithuanian study with the same outcome measure showed a lifetime Pap smear uptake increase in 2006–2014 from 73.7 to 86.1% [13]. The increase in Estonia can be explained by a national CC screening program established in 2006, which most likely has positively impacted the CC awareness and therefore urged the Pap smear uptake. On the other hand, it is surprising that in 16 years the organized CC screening program has not had an effect on CC incidence [7]. Furthermore, the stage distribution has shifted towards later stages, and the mortality trend has decreased only slightly [7]. These results reflect the ineffectiveness of the nation-wide screening program and should be investigated further.

Despite the existence of uniform population-based CC screening, the lifetime uptake of Pap smear was significantly lower among non-Estonians. The found inequality is consistent with the results of other international studies [14–19], showing that being of foreign origin has had a significant impact on the probability of not attending screenings. While in 2004, ethnicity was not a significant predictor of Pap smear uptake in Estonia [10], we may assume that the inequality has increased, as we observed a more modest increase in Pap smear uptake among non-Estonians than among Estonians. Although most of non-Estonians are originating from the immigration more than 30 years ago, previous studies have shown that non-Estonians have poorer health indicators, poorer self-rated health, shorter life expectancy [20–24], and they are less aware of the screening programs than Estonians [25], making it essential to identify non-Estonians’ barriers to attend, and to facilitate interventions to increase the participation of minority groups.

As consistent with previous studies [13, 15, 18, 26–28], being married, living with a partner, and being widowed, separated or divorced, was a stronger predictor for uptake of a Pap smear. Although all target group women in Estonia are invited to CC screening in every 5 years, a Pap smear is also offered to women as part of pre- or post-natal services during their visit to gynaecologist or midwives, which puts women in a sexual relationship in a more favourable position to have an opportunistic Pap smear. Single women may underestimate their risk of CC or other gynaecological conditions and therefore not feel the need for regular check-ups showing the urgent need to improve the organized screening.

The results of this study confirmed the positive effect of education on increased Pap smear uptake, as found in many other studies [14–16, 18, 26, 29]. There might be several explanations: well educated individuals have greater awareness about their health risks, more knowledge about health issues, as well as a better access to information and resources for health improvement [30]. Although in this study, lifetime uptake of the Pap smear was found to be significantly higher among women with secondary and higher education, also was observed that the gap between education levels decreased from 2004 to 2020 (Fig. 2D).

Unemployed women were less likely to undergo a Pap smear test, consistent with findings from both a 2004 Estonian study [9] as well as studies from other countries [16–18, 27, 28]. Lack of health insurance seems to be the most obvious reason for this finding but according to our study, having health insurance was only associated with the lifetime uptake of Pap smear in univariate analysis and after adjusting to other variables this association disappeared.

Being of normal weight or less was found to be an important positive predictive factor of Pap smear uptake in this study, as has similarly been described in a previous Estonian study [11] and in a meta-analysis [31]. Therefore, particular attention should be paid to overweight as well as to obese women as these groups have a worse prognosis for CC. Possible barriers for testing might be embarrassment in the examination room, negative reactions from healthcare providers, lectures about weight, and inadequate equipment for larger women [32]. To minimize these barriers for overweight women, an alternative could be offering them an HPV self-sampling test. An Estonian pilot study recently showed the feasibility and positive acceptance of HPV self-sampling among long-term CC screening of non-attendees [33] which might also increase the participation of obese women.

Women with chronic illness were more likely to undergo a Pap smear test compared to women without chronic illness. Because of their diagnosis they might go more regular health checks and during these visits they can be encouraged to participate in screenings also.

A more unexpected result was that women feeling depressed in the past 30 days were more likely to undergo a Pap smear test compared to women not feeling depressed. This result differs from other studies where unhappy and depressed women reported having a lower likelihood of cancer screening [15, 28, 34] but it can arise from differences in research methodology and scales of measuring depression and this result should be considered with caution.

Unhealthy lifestyle choices were associated with Pap smear uptake. Not being physically active during leisure time or not being able to exercise due to any health condition were found to be strong predictors of not undergoing a Pap smear test, as found in other studies [13, 15, 16, 35] including in one Estonian study [10]. In accordance with similar studies, our findings showed that daily smokers were less likely to use Pap smear testing than never smokers [10, 26–28]. Smoking is a risk factor for both CC and the HPV that causes it, so if smoking increases the chances of not getting a Pap smear for early detection of CC, the risks accumulate [36]. Being physically active and non-smoking are part of a person’s health behaviour like taking care of one’s health and attending a screening.

Based on our results, an effective cervical cancer prevention policy in Estonia should focus on non-Estonian, single, less educated, unemployed, overweight and obese, physically inactive and daily smoking women.

The main strength of this study is the use of a nationally representative sample and a long study period. The methodology of the survey, which started already in 1990, has been maintained the same and therefore the results of different years are comparable. It was also considered having self-reported data as a strength as it gives a valuable insight into women’s lifestyle and preferences over the course of 16 years and includes Pap smears taken by private health care providers. At the same time, it was acknowledged that the self-reported information might have led to an overestimation of adherence to cancer screening [37, 38]. In addition, while it would have been more suitable to measure timely Pap smear uptake (3- or 5-year interval) lifetime uptake of Pap smear was used instead, as there was no data in such detail from 2004 survey. At the same time, by omitting the 2004 survey completely, valuable baseline information about the pre-screening era would be lost, which would have narrowed the results of this study. Furthermore, it was suspected that not all women knew what a Pap smear is or what kind of tests are taken during a regular health check since it was necessary to exclude 3.7% of women who did not answer the question about giving a Pap smear and one third of respondents who did not answer about the initiator (doctor, screening or woman herself) of their most recent Pap smear (data not shown).

## Conclusions

Although the reported lifetime uptake of Pap smear increased significantly during 2004–2020, sociodemographic, socioeconomic, health-related and lifestyle inequalities were found. Lower lifetime uptake of Pap smear was associated from sociodemographic factors with younger age, being non-Estonian and single, from socioeconomic factors with lower educational level and unemployment, from health indicators with higher body mass index indicating overweight and obesity, presence of chronic disease and depressiveness, from lifestyle factors with non-smoking. From the public health view the found inequalities in Pap smear uptake indicate an opportunity for development of targeted CC prevention strategies to in order to achieve reduction in the prevalence and mortality rates of CC in Estonia.

## Data Availability

Primary data of the Health Behaviour among Estonian Adult Population Study is not publicly available but it can be requested from the National Institute for Health Development by sending an application to Rainer Reile.
